# Gut microbiota: a potential new regulator of hypertension

**DOI:** 10.3389/fcvm.2024.1333005

**Published:** 2024-06-27

**Authors:** Yanmin Ge, Jiaxin Wang, Lincong Wu, Junduo Wu

**Affiliations:** ^1^ Department of Cardiology, The Second Hospital of Jilin University, Changchun, Jilin, China; ^2^ Department of Cardiology, The Second Affiliated Hospital of Chongqing Medical University, Chongqing, China

**Keywords:** gut microbiota, hypertension, short-chain fatty acids, lipopolysaccharide, blood pressure

## Abstract

Hypertension is a significant risk factor for cardiovascular and cerebrovascular diseases and has become a global public health concern. Although hypertension results from a combination of factors, the specific mechanism is still unclear. However, increasing evidence suggests that gut microbiota is closely associated with the development of hypertension. We provide a summary of the composition and physiological role of gut microbiota. We then delve into the mechanism of gut microbiota and its metabolites involved in the occurrence and development of hypertension. Finally, we review various regimens for better-controlling hypertension from the diet, exercise, drugs, antibiotics, probiotics, and fecal transplantation perspectives.

## 1 Introduction

Hypertension is the most important risk factor for cardiovascular and cerebrovascular diseases and often coexists with other risk factors, which in turn leads to other serious diseases such as heart, brain, and kidney. The number of hypertensive patients has doubled over the past 30 years. However, only 13% of hypertensive patients are effectively controlled (1, 2). The gastrointestinal tract plays a crucial role in regulating the relationship of the external environment to commensal and/or pathogenic substances such as food and bacteria that communicate with the human host. It is an entry point for many harmful environmental risk factors for hypertension (3). A decrease in gut microbiota abundance, diversity, and an increase in the Firmicutes (F)/Bacteroidetes (B) ratio increase the risk of hypertension (4). Miao et al. (5) provided convincing evidence of a causal relationship between gut microbiota and blood pressure by using Mendelian randomization. An imbalance in gut microbiota can lead to hypertension and is closely associated with short-chain fatty acids (SCFAs), trimethylamine oxide (TMAO), hydrogen sulfide (H_2_S), and lipopolysaccharide (LPS), which are metabolites produced by gut microbiota. This article provides an overview of the mechanism by which gut microbiota contributes to the development and progression of hypertension, as well as the impact of gut microbiota metabolites on hypertension. Additionally, the article discusses better options for controlling hypertension, including diet and exercise, medication, and fecal transplantation.

## 2 The composition of gut microbiota in the host

In healthy adults, the composition of the gut microbiota is stable and consists of trillions of microorganisms, but mainly four species: (1) Firmicutes, (2) Bacteroidetes, (3) Actinobacteria, and (4) Proteobacteria. Changes in the proportion of Firmicutes (F) and Bacteroidetes (B) microbial communities, i.e., F/B ratio, can be used as biomarkers of pathological conditions (6). *α* diversity and *β* diversity are commonly used indicators to evaluate microbiota ecology, with higher *α* diversity indicating a greater number of community species and *β* diversity indicating the number and distribution of species in different communities (7). Imbalanced gut microbiota may result from factors such as improper diet, stress, medication, age, infection, pH changes, and immune dysfunction.

## 3 The composition of gut microbiota in cardiovascular disease

There is growing evidence of an association between cardiovascular disease and changes in the abundance or diversity of gut bacteria (Table 1). A cross-sectional study found that the gut microbiota in chronic heart failure is characterized by high compositional changes, low bacterial abundance, and a decrease in butyrate-producing bacteria, changes that may be associated with chronic immune activation (8). Another study, which involved 161 patients with coronary heart disease and 40 controls, showed that the structural features of the gut microbiota changed with the development of coronary heart disease. The abundance of Haemophilus and Klebsiella increased with the severity of coronary heart disease compared to healthy controls (9). In the standard mouse model of myocardial infarction (MI), the production of lactic acid bacteria and short-chain fatty acids is reduced after myocardial infarction, and supplementation with lactic acid bacteria or short-chain fatty acids will improve cardiac function (10). In a study of 30 atrial fibrillation (AF) and 30 healthy controls, it was found that there was a significant decrease in intestinal bifidobacteria, an increase in the abundance of Lactobacillus, Clostridium, Haemophilus, and a decrease in isovaleric acid and isobutyric acid in the AF group (11). All the above studies suggest the involvement and important regulatory role of the gut microbiota (GM) in cardiovascular diseases.

**Table 1:** Changes in gut microbiota in cardiovascular diseases

Row	Disease	Year	Subjects	Microbiome changes	Metabolites changes	Findings	Ref
1	Stable systolic Heart failure	2018	*N *=* *84 HF *N *=* *266 Controls	↑Prevotella Hungatella Succinclasticum ↓Lachnospiracea Blautia Eubacteriumhalli Ruminococcaceae Faecalibacterium Bifidobacteriaceae	Not reported	Butyrate production genetic potential is lower in HF microbiome	(6)
2	Coronary artery disease (CAD)	2019	161 CAD patients and 40 healthy controls	↑Veillonella Haemophilus Klebsiella ↓Lachnospiraceae Ruminococcaceae	Not reported	Certain bacteria might affect atherosclerosis by modulating the metabolic pathways of the host	(7)
3	Myocardial infarction (MI)	2019	MI C57BL/6J mice	↓Lactobacillus	↓acetate butyrate propionate.	Gut microbiota-derived SCFAs play an important role in maintaining host immune composition and repair capacity after MI	(8)
4	Atrial Fibrillation (AF)	2023	*N *=* *30 AF *N *=* *30 sinus rhythm (SR)	↑Lactobacillus Fusobacterium Haemophilus ↓Bifidobacterium	↓isobutyric acid isovaleric acid	GM and SCFA dysbiosis might play a crucial part in the occurrence and development of AF.	(9)
5	Hypertension	2017	*N *=* *60 hypertension *N *=* *60 healthy controls	↑Klebsiella Clostridium Streptococcus Parabacteroides Eggerthella Salmonella ↓Faecalibacterium Roseburia Synergistetes	↑LPS	Specific changes in microbial diversity, genes, species, and functions of the gut microbiome in hypertensive patients	(10)
6	Hypertension	2019	529 participants of the biracial (African- and European-American)	↑Robinsoniella Catabacter ↓Sporobacter Anaerovorax	Not reported	Gut microbial diversity was inversely associated with both hypertension and systolic blood pressure.	(11)
7	Hypertension	2015	*N *=* *5 WKY *N *=* *6 SHR	↑Streptococcus Turicibacter lactate-producing bacteria ↓Coprococcus Pseudobutyrivibrio	↓Acetate butyrate lactate	Hypertension is associated with gut microbiota dysbiosis.	(12)

Hypertension induces changes in the intestinal environment, which can induce dysbiosis in internal microbial communities. The hypertensive group was found to have significantly reduced microbiota richness, evenness, and diversity (12). In a cross-sectional analysis, the composition of the gut microbiota was found to be significantly different in terms of blood pressure, and gut microbial diversity was negatively correlated with systolic blood pressure (13). Hypertensive patients have an increase in harmful genera with pro-inflammatory responses and reduced immunity in the intestine, such as Klebsiella, Streptococcus, and Parabacterium, which may be key genera leading to the development of hypertension; while short-chain fatty acid producers are relatively reduced (14). Subsequent analysis of sixty hypertensive patients and sixty matched controls revealed reduced gene counts, reduced diversity, and altered microbial composition, consistent with previous findings (12).

In addition, another study showed differences in GM between males and females. Virwani et al. (15) found *β* diversity and GM composition were significantly different only in female hypertensive patients, as shown by a significant increase in the number of Ruminococcus gnavus, Clostridium bolted, and Bacteroides ovale, which was not observed in male hypertensive patients. These differences may be related to hormone secretion in men vs. women. In a large study of 4,672 subjects from six different ethnicities, HEalthy Life (HELIUS) in the urban setting showed that gut microbiota composition and blood pressure (BP) were closely correlated, despite large differences between age and ethnic subpopulations (16). All of the above studies illustrate the fact that factors such as racial subgroups and gender affect the composition of the gut flora.

## 4 Destruction of the intestinal barrier in hypertensive

In a healthy state, intestinal barrier function is maintained by physical factors, including tight junctions between epithelial cells, mucus production, and mucosal immunity. When the intestinal barrier function is normal, the intestinal permeability is low, which can effectively inhibit the leakage of intestinal pathogens, intestinal endotoxin, and other substances into the body. Abnormal changes in intestinal permeability and structural damage to the intestinal mucosa lead to the translocation of bacteria and toxic products into the blood circulation, causing the development of systemic inflammation (17). Chronic inflammation can perpetuate the hypertensive state, exacerbate hypertensive target organ damage, and promote the development of resistant hypertension (18).

Several studies have shown that hypertensive patients have significant disturbances in the gut microbiotaand intestinal barrier dysfunction (19–21). In hypertension, the intestinal barrier is incomplete due to disturbance of microbial composition, causing mucin degradation, transmission and adhesion of pathogens and pro-inflammatory LPS, followed by activation of resident immune cells that trigger the inflammatory cascade, which leads to intestinal dysbacteriosis (22). In addition, intestinal fatty acid binding protein, lipopolysaccharide, and enhanced gut-targeted pro-inflammatory T helper cells (Th) 17 were significantly increased in plasma, and zonulin (intestinal epithelial tight junction protein regulator) was significantly increased in hypertensive patients, suggesting increased intestinal permeability and barrier dysfunction secondary to intestinal inflammatory responses (23). Similar studies have found that spontaneously hypertensive rats (SHR) showed reduced intestinal mucosal thickness and blood flow, decreased glandular goblet cells, decreased intestinal villus height, decreased tight junction protein, and increased intestinal permeability, suggesting that hypertension can lead to impaired intestinal barrier function (19).

## 5 The key mechanisms of gut microbiota regulating the occurrence and development of hypertension

### 5.1 Regulation of gut microbiota in inflammation mediated hypertension

Inflammation is the body's defense response to injury or infection, but excessive inflammation can lead to disease. Studies have shown that people with high blood pressure often exhibit higher levels of inflammation than healthy people. Therefore, inflammation may be one of the causes of increased blood pressure. Gut microbiota imbalance induces immune disorders, which cause chronic inflammatory responses and induce endothelial dysfunction, thereby causing increased blood pressure. Karbach et al. (24) found that gut microbiota may contribute to the development of hypertension *in vivo* by promoting angiotensin II (Ang-II) induced monocyte chemoattractant protein 1 (MCP-1)/IL-17-driven vascular immune cell infiltration and inflammation.

Both obstructive sleep apnea (OSA) syndrome and a high-salt diet contribute to the development of high blood pressure. OSA leading to hypertension is associated with dysbacteriosis of the gut microbiota by significant loss of anti-inflammatory T regulatory cells (Tregs) and increases in pro-inflammatory TH1 and TH17 cells in the ileum, cecum, and brain (25). In addition, the researchers found that rats with OSA combined with a high-salt diet released Th1-related cytokines (IFN-γ), inhibited anti-inflammatory cytokines (TGF-β1) to increase blood pressure, and affected the gut microbiome (26). Some studies have reported that a high-salt diet can inhibit lactic acid bacteria leading to gut microbiota disturbance and promoting the production of pathogenic TH17 cells, thereby promoting salt-sensitive hypertension (27). Ferguson et al. (28) found that mice fed a high-salt diet exhibited increased intestinal inflammation, including a significant increase in the B7 ligand CD86, and that the formation of IsoLG protein adducts in CD11c + bone marrow cells, leading to hypertension. These observations suggest that gut microbiota-targeted therapy can be used as a new strategy for the prevention and treatment of OSA and salt-sensitive hypertension, and its underlying mechanism is the direction of further research.

### 5.2 Gut microbiota drives hypertension through the brain-gut axis system

The brain-gut axis is a bidirectional communication pathway composed of the central nervous system the enteric nervous system and the autonomic nervous system, and gut microbiota imbalance causes increased inflammation, which further leads to autonomic disorders, while autonomic disorders affect the intestinal flora (29, 30). The gut microbiota mediates and regulates the release of gut peptides, which can signal to the brain via the vagus nerve and the brain can also be counter-regulated (31). Hypothalamic-driven increases in intestinal sympathetic tone, intestinal pathology, and inflammatory interactions exacerbate elevated blood pressure (32). Some studies have found that when the gut microbiota is imbalanced and causes intestinal barrier disruption, its metabolites act on the nerves of the intestinal wall and activate the central sympathetic nerves, resulting in systemic arteriolar contraction and hypertension (33). Metabolites of the microbiota have been found to readily cross the blood-brain barrier, causing central inflammatory symptoms in the brain and leading to hypertension (34).

Santisteban et al. (19) found that disturbed gut microbiota in SHR enhanced sympathetic neuronal communication between the gut and the hypothalamic paraventricular nucleus (PVN). However, after the transplantation of gut microbiota from healthy rats into hypertensive rats, inflammation and sympathetic stimulation in the PVN were reduced, and blood pressure was steadily decreased. This suggests that gut microbiota metabolism affects enhanced information transmission in the intestine and sympathetic nervous system (SNS), and SNS activation can also in turn affect gut microbiota metabolism regulate dopamine and norepinephrine secretion, and affect blood pressure.

Torl et al. (35) found that intestinal dysbiosis was associated with sympathetic outflow via stimulation of NADPH-oxidase-derived ROS in the brain in SHR, and these central effects may be associated with reduced expression of butyrate-sensitive receptors in the hypothalamus, Th17, and macrophage infiltration in the hypothalamic PVN, and higher plasma levels of LPS. The immunosuppressive agent mycophenolate mofetil (MMF) inhibits neuroinflammation in PVN, increases the proportion of Tregs in mesenteric lymph nodes and Th17 and Th1 infiltration in the aorta, normalizes the gut microbiota, improves aortic endothelial function, and reduces systolic blood pressure (36). Taken together, this suggests that the gut microbiota can stimulate sympathetic drive, possibly through direct intestinal wall nerve-brain interactions or by promoting neuroinflammation and promoting the development of hypertension (37).

### 5.3 Gut microbiota affects hypertension through lipid metabolism pathways

Hypertension and dyslipidemia tend to coexist, while current studies have shown that dyslipidemia plays an important role in the mechanism of hypertension (38, 39). Normal gut microbiota regulates lipid metabolism homeostasis, and when gut microbiota is disturbed, it leads to abnormal lipid metabolism and promotes the formation of hypertension. Chronic intake of a high-fat diet has been found to alter the diversity of gut microbes and the structure of the microbiota, which may further alter cholesterol homeostasis in mammals (40). Ding et al. (41) found that mice fed a high-fat diet developed lipid metabolism disorders, gut microbiotaimbalance, a significant increase in the number of Firmicutes, and a decrease in the number of Bacteroides, resulting in increased free radicals in the body, causing oxidative stress and lipid peroxidation, and at the same time, a high-fat diet resulted in destruction of the submucosal epithelium, neutrophil infiltration, and loss of tight junction proteins (42). These changes are similar to the characteristics of the gut microbiotaof hypertension. The latest guidelines for the management of hypertension suggest that high cholesterol levels are an important modifiable risk factor for hypertension (43). So modulating lipid metabolism can be used as a new way to prevent hypertension.

## 6 Metabolites of gut microbiota and blood pressure regulation

**Figure 1:**
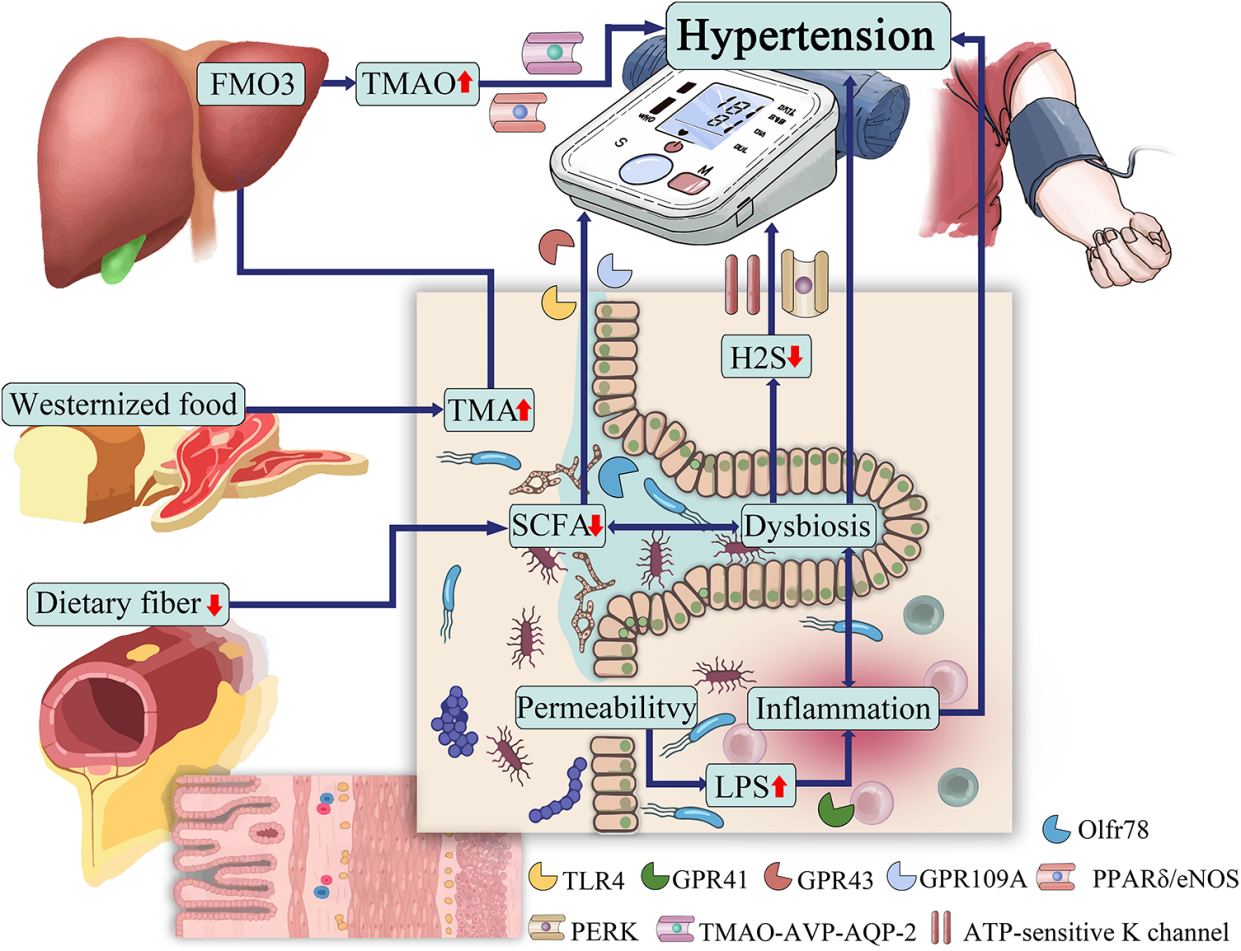
The role of gut microbiota in hypertension.

The gut microbiota can produce a range of bioactive metabolites, such as enzymes, peptides, antibiotics, amino acids, hormones, and vitamins, which can mediate host receptor activation, signaling, and immunomodulatory effects (44). Among them, SCFAs, TMAO, H_2_S, and LPS are closely related to the development of hypertension (45). Hypertensive patients have abundant gut microbial enzymes involved in TMAO production, while enzymes producing SCFAs are reduced, LPS has a pressor effect, and H_2_S can reduce blood pressure (46). The gut microbiota and its metabolites are involved in the development of hypertension (Figure 1).

### 6.1 SCFAs

SCFAs are short-chain fatty acids with chain lengths ranging from 1 to 6 carbon atoms, produced by microbial fermentation of dietary carbohydrates.SCFAs are metabolites produced by microbial fermentation of dietary carbohydrates and are currently the most well-studied among gut microbial metabolites. Of all these short-chain fatty acids in fermentation products, acetate (C2), propionate (C3) and butyrate (C4) account for about 80% of all short-chain fatty acids. In the human gut, phylum Bacteroidetes mainly produce acetate and propionate, while Firmicutes mainly produce butyrate (47). These short-chain fatty acids regulate blood pressure by dilating blood vessels (48).

Increasing evidence suggests that SCFA levels and abundance of intestinal bacteria are lower in hypertensive patients than in normotensive people. It has been found that the abundance of SCFAs-producing bacteria Faecalibacterium prausnitzii, and Roseburia hominis were lower in hypertensive population, whereas the abundance of Bacteroides coprocola, Bacteroides plebeius and genera of Lachnospiraceae were higher. SCFAs showed antagonistic effects in both plasma and feces, and fecal levels of SCFAs were detected to be Significantly higher levels of SCFA in feces, and significantly lower levels of SCFA in plasma were detected in the hypertensive population, possibly due to less efficient SCFA absorption in the hypertensive population (49). A 5-year follow-up study of a cohort of 26 patients showed that SCFAs (including acetate, propionate, and butyrate) in the stool of hypertensive patients were higher than those in normotensive patients, and were significantly associated with 24-h mean blood pressure in both genders (50). Another study in patients with essential hypertension also confirmed that fecal SCFA excretion was positively correlated with blood pressure (51).

SCFAs act as ligands and bind to host microbial metabolites G protein-coupled receptors (GPCRs) to regulate blood pressure, including GPR41, GPR43, GPR109A, and olfactory receptor 78 (Olfr78) (52). Depending on the length of their aliphatic tails, receptors exhibit different affinities for short-chain fatty acids. GPR41 and Olfr78 prefer to bind to acetate and propionate, while GPR43 binds propionate, butyrate, and acetate with lower affinity, and GPR109a binds mainly butyrate (53). GPR41 expression sites are mainly located in the vascular endothelium, and GPR43 is more prevalent in immune cells. GPR109A is widely expressed in white and brown adipose tissue, keratinocytes, and various immune cells (48). Compared with wild-type mice, GPR41, GPR109A and GPR43/109A knockout mice had higher diastolic pressure and pulse pressure, and the degree of peripheral vascular fibrosis increased (54). Pluznick et al. (55) found that Olfr78 distributed in vascular smooth muscle affects renin secretion and increases blood pressure by acting on renal afferent arterioles, SCFAs regulate renin release through afferent arterioles, resulting in increased blood pressure, and Gpr41 has an antagonistic effect. The above studies suggest that short-chain fatty acids affect blood pressure differently depending on the receptor involved.

SCFAs also exert potent anti-inflammatory effects by regulating the activity of immune cells, thereby reducing the damage of hypertension to target organs. Oral administration of acetate and butyrate to hypertensive rats inhibited the vascular LPS/TLR4 pathway, increased the infiltration of Treg cells into the vascular system, and reduced the F/B ratio (56). In TLR7-induced systemic lupus erythematosus mice, acetate and butyrate enhanced intestinal integrity, reduced endotoxemia, and reduced Th17 infiltration in the vascular wall, rebalanced the intestinal immune system, reduced endothelial dysfunction, and prevented the development of hypertension (57). Studies have shown that propionate attenuates the hypertensive immune inflammatory response by inhibiting the expression of CD4+ T cells, CD8+ T cells, and Th17 in hypertensive mice (58). Propionate and butyrate supplementation therapy can reduce the inflammatory response and exert antihypertensive effects by promoting autophagy and M2 polarization of placental bed macrophages in rats with preeclampsia (59). Short-chain fatty acids may also be activated by reducing the CoII oxidase-intracellular reactive oxygen species pathway, interfering with gut-neuronal communication in the paraventricular nucleus of the hypothalamus, or directly reducing norepinephrine production, thereby inhibiting sympathetic hyperactivation (35). Besides, Wang et al. (60) showed that sodium butyrate exerts an anti-Ang II-induced hypertensive effect by inhibiting the renin-angiotensin system mediated by the renin receptor (PRR). The above indicates the involvement and therapeutic potential of SCFAs in hypertension. It is important to note that the pharmacokinetics of SCFAs (e.g., production and tissue utilization) vary widely between humans and smaller animals, which limits the direct extrapolation of SCFAs peripheral effects from animal models to human disease (61).

### 6.2 TMAO

TMAO is a metabolite and bioactive molecule whose precursor is TMA (62). TMAs come directly from foods rich in choline, L-carnitine, and phosphatidylcholine, such as red meat, salted fish, eggs, and dairy products (63). TMA is mainly absorbed into the circulation and then oxidized to TMAO by the hepatic rate-limiting enzyme heparin monooxygenase 3 (FMO3).

TMAO is small in size and hydrophilic and hydrophobic at the same time, and manifests itself as a chaotropic agent capable of altering protein conformation and possibly acting as an allosteric modulator of proteins, for example, influencing intracellular protein unfolding or endoplasmic reticulum stress responses (64). It has been found that TMAO promotes Ang II-induced vasoconstriction and thereby hypertension, which is associated with the protein kinase R (PKR)-like endoplasmic reticulum kinase (PERK)/reactive oxygen species (ROS)/Ca2+/calmodulin-dependent protein kinase II (CaMKII)/phospholipase Cβ3 (PLCβ3) axis (65). Ufnal et al. (66) speculated that TMAO et al. could enhance protein folding and ligand binding, thereby affecting the structure of Ang II and prolonging the effect of increasing blood pressure. TMAO may also be involved in tissue osmotic pressure in vertebrates. It has been found that the increase of plasma TMAO levels in SHR leads to higher plasma osmolality, triggers the regulation of TMAO-AVP-AQP-2 in SHR, causes greater water reabsorption, and ultimately leads to hypertension (67). In addition, TMAO increases the risk of hypertension by promoting vascular endothelial dysfunction (68). The above studies illustrate that hypertension has a mutually promoting relationship with TMAO.

Several studies have shown a significant correlation between TMAO concentrations and the risk of hypertension (69, 70). People with high TMAO concentrations had a 12% increased risk of hypertension compared to those with low circulating TMAO concentrations (71). Nie et al. (72) found that higher TMAO levels in serum were associated with an increased risk of first stroke in hypertensive patients. However, it has also been found that chronic low doses of TMAO can reduce diastolic dysfunction in the heart of hypertensive rats (73). Therefore, the effect of TMAO on hypertension remains to be further explored.

### 6.3 LPS

LPS is a component of the outer membrane of gram-negative bacteria and is composed of lipids and sugars (74). In healthy subjects, the gut-blood barrier prevents LPS from entering the circulating bloodstream. However, leakage of the gut-blood barrier due to ecological dysregulation leads to LPS entering the bloodstream (75). Toll-like receptor 4 (TLR4) is the membrane receptor of LPS, which, when activated, triggers NF-*κ*B signaling and produces pro-inflammatory cytokines to release TNF-α, IL-1, IL-6, interferon, etc., further damaging intestinal mucosal function and increasing intestinal permeability (76).

In human studies, plasma LPS concentration was positively correlated with hypertension, LPS stimulated and increased the expression of TLR4, releasing inflammatory factors to promote the occurrence of hypertension (56, 77). Dange et al. (78, 79) found that LPS administration to rats increased heart rate and norepinephrine levels, decreased baroreflex sensitivity, increased neuroinflammation, and increased TLR and TNF-α expression in PVN, thereby increasing hypertension. If TLR4 is inhibited in PVN, sympathetic activity is inhibited, and then blood pressure is reduced. Some studies have found intestinal dysbacteriosis in patients with preeclampsia, and their plasma LPS is higher than that in healthy controls (80). The above studies show that LPS increases blood pressure by promoting the inflammatory response.

### 6.4 H_2_S

Intestinal microbial fermentation also produces gaseous compounds, the main component of which is hydrogen sulfide (H_2_S), which has physiological functions such as improving endothelial dysfunction and alleviating vascular oxidative stress (81). Tomasova et al. (82, 83) found that H_2_S may help to reduce blood pressure, and if H_2_S synthesis is dysregulated it promotes the occurrence of hypertension. H_2_S is an endogenous vasoactive factor that causes concentration-dependent vasodilation to exert a hypotensive effect, and its mechanism may be related to the activation of ATP-sensitive K channel opening (84, 85). It has been found that in AngII-induced hypertension mouse models, the addition of NaHS treatment reduces blood pressure (86). In addition, H_2_S plays a protective role in renal artery endothelium in hypertensive patients by activating the peroxisome proliferator-activated receptor δ/endothelial nitric oxide synthase (PPARδ/eNOS) pathway to activate the pathway (87). The antihypertensive effect of H_2_S has been confirmed, but its specific mechanism still needs further exploration, and H_2_S may be applied in the clinical treatment of hypertension in the future.

## 7 The therapeutic potential of targeting the gut microbiota in hypertension

**Figure 2:**
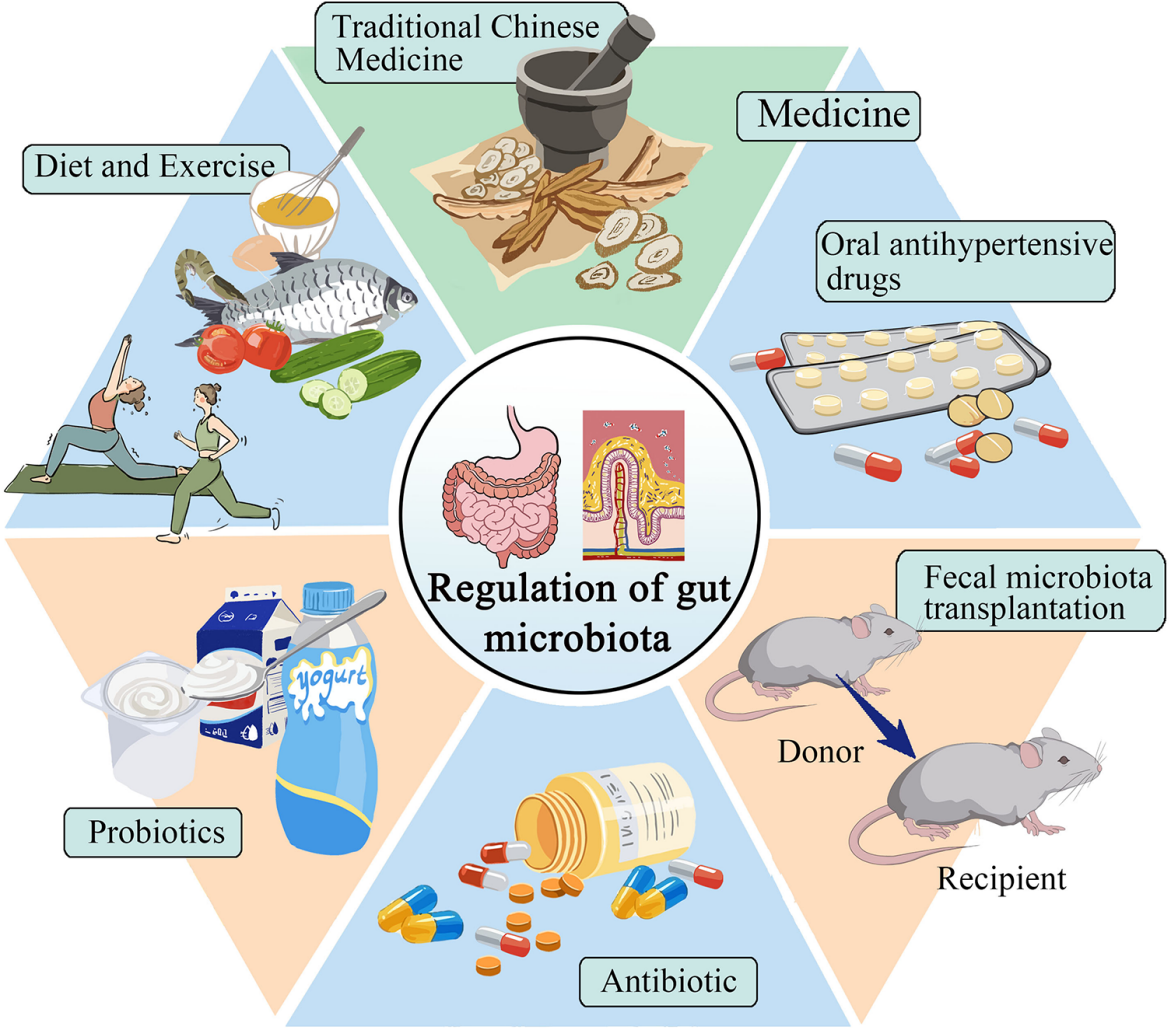
Selection of treatment plans for gut microbiota in patients with hypertension.

Improving or reversing gut microbiota has become a hot topic in treating hypertension. This includes exercise, dietary regulation, probiotic supplements, antibacterial interventions, medication, and fecal microbiota transplantation (Figure 2).

### 7.1 Diet and exercise

For patients with hypertensive cardiovascular diseases, non-drug prevention strategies, diet, and exercise are currently the most direct and easy ways to prevent and treat hypertension. By changing dietary habits, people can alter the gut microbiota and protect the body, helping to regulate blood pressure. Studies have shown that a high-fiber diet can enhance gut health, increase the presence of acetate-producing bacteria, and effectively reduce both systolic and diastolic blood pressure in hypertensive mice (88). The Mediterranean diet is a nutritionally recommended dietary pattern that includes high consumption of cereals, fruits, vegetables, and legumes (89). Studies have shown that the gut microbiota of people on the Mediterranean diet can prevent the onset of chronic non-communicable degenerative diseases and reduce all-cause mortality (90). Choo et al. (91) found that compliance with a Mediterranean diet supplemented with dairy decreased blood pressure in high experimenters. It has been found that daily consumption of vitamin C reduces blood pressure in SHR and can improve the diversity and abundance of gut microbiota, facilitate the recovery of intestinal mucosal integrity, and reduce inflammatory response and oxidative stress (92). In a cross-sectional study, it was determined that food polyphenolic compounds induced changes in intestinal bacterial composition to influence SCFA production and absorption, which in turn affected blood pressure. All of the above indicate that dietary habits can regulate gut microbiota and lower blood pressure (93). Intermittent fasting has been found to lower blood pressure by altering gut microbiota and thereby normalizing bile acid signaling (94). The above different dietary habits can reduce blood pressure by changing the gut microbiota, and an individualized diet can be developed to treat hypertension in the future.

Moderate exercise has numerous benefits on the gut microbiota, including promoting changes in its structure and state, producing beneficial metabolites, regulating the immune response system, and helping to prevent and control hypertension. According to a study conducted by Xia et al. (95), exercise training for male SHRs resulted in a sustained decrease in systolic blood pressure. Furthermore, the study reported a decrease in the number of activated microglia, an improvement in neuroinflammation, intestinal pathology, inflammation, and permeability of PVN. These improvements may be associated with the enrichment of probiotics. Liuzijue training is a traditional exercise that combines breathing meditation with physical exercise. This exercise can transform the structure of the gut microbiota in hypertensive patients to that of healthy individuals (96). Relative increases in fecal metabolites (e.g., microbially produced acetic acid, propionate, and butyrate) are associated with enhanced overall health in athletes compared with sedentary individuals (97). The above shows that exercise can affect the gut microbiota and effectively reduce blood pressure. Meanwhile, exercising for long periods can yield significant and long-term benefits.

### 7.2 Medication

#### 7.2.1 Oral antihypertensive drugs

Oral antihypertensive drugs have an immediate effect on the treatment of hypertension and are currently the preferred method for the control of hypertension. Some studies (98) have found that captopril affects the structure and composition of the intestinal microbiota, and improvements in intestinal pathology and permeability, reduced fibrosis area, increased goblet cells, increased villus length, reduced neuroinflammation, and prolonged antihypertensive effects after discontinuation have been observed, the mechanism of which may be related to the brain-gut axis. Wu et al. (99) found that candesartan protected ileal and colonic pathology in SHR, prevented hypertension-related intestinal barrier damage, increased microbial production of SCFAs, and retained intestinal lactobacilli under hypertensive conditions. Lactobacillus may contribute to its ability to reduce inflammatory cytokines and oxidative stress, protect intestinal integrity and increase the production of short-chain fatty acids (100). In addition, losartan has been found to reduce sympathetic activity in the colon, reduce the intestinal F/B ratio, protect the intestinal mucosa, and lower blood pressure losartan has been found to decrease colonic sympathetic activity, increase intestinal integrity, and decrease blood pressure (101, 102). Spironolactone also has the above-mentioned antihypertensive mechanism (103). Oral antihypertensive drugs provide new ideas for the treatment of hypertension by regulating blood pressure through gut microbiota.

#### 7.2.2 Traditional Chinese medicine treatment

In recent years, several studies have shown that traditional Chinese medicine may intervene in hypertension treatment through gut microbiota. Baicalin repairs intestinal barrier damage, promotes the expression of tight junction proteins, and promotes the level of butyric acid-producing bacteria to participate in the antihypertensive mechanism (104). Wang et al. (105) found that berberine decreased the proportion of F/B, increased the abundance of lactic acid bacteria, inhibited the production of TMAO in hypertensive mice, and decreased blood pressure in Ang II-induced hypertensive rats. Sanoshashinto contains baicalin and berberine, which can dilate blood vessels, protect the endothelium, reduce left ventricular hypertrophy, change the intestinal microbiota, and have a comprehensive antihypertensive effect (106). The antihypertensive effect of curcumin is related to the brain-gut axis, it increases the level of butyrate in the serum of SHR rats, activates GPR43 in PVN, improves the dysregulation of the brain-gut axis, thereby increasing the length of goblet cells and villi, and restores the mRNA levels of tight junction protein 1 and occlusin in the intestine (107). Su et al. (108) used Polygonatum sibiricum Red. superfine powder to inhibit LPS-induced activation of TLR4/MyD88 signaling in blood vessels, improve vascular endothelial function, regulate gut microbiota structure, and then reduce metabolic hypertension. Zhengan Xifeng decoction can change the proportion of SCFAs produced in SHRs, repair the damaged intestinal mucosa, and reduce D-lactate, diamine oxidase, and other inflammatory factors in blood circulation, thereby reducing blood pressure (109).

Currently, two main issues need to be addressed regarding traditional Chinese medicine and hypertension. Firstly, the antihypertensive mechanism of traditional Chinese medicine is not yet fully understood. Secondly, there is a lack of substantial clinical data to support the relationship between traditional Chinese medicine and hypertension. Therefore, it is necessary to conduct a large number of basic experiments along with clinical studies to provide more definitive information in this area.

### 7.3 Probiotics

Probiotics are live microorganisms that exert beneficial effects on the body, and may exert antihypertensive effects by regulating gut microbiotametabolites, improving oxidative stress, and reducing chronic inflammation of blood vessels. A meta-analysis involving 2,037 participants showed that the consumption of probiotics SBP reduced by 3.05 mmHg and DBP by 1.51 mmHg, indicating that probiotics were effective in lowering blood pressure (110). According to Khalesi et al. (111) found that the intake of probiotics could moderately improve blood pressure, and the effect was better when the type of probiotics ingested increased and the intake time was prolonged.

On the one hand, the engineered probiotic (CB-GPL-1) participates in maintaining intestinal homeostasis by up-regulating the abundance of Lactobacillus and down-regulating the level of Porphyromonas, and on the other hand, it is mediated by glucagon-like peptide-1 (GLP-1) and butyric acid, regulates the RAAS system and GPR109A in the kidney and initiates the antihypertensive mechanism, and activates the AMPK signaling pathway to regulate myocardial proliferation and apoptosis, and improves myocardial cell hypertrophy and ventricular wall fibrosis (112). Kong et al. (113) found that the antihypertensive effect of probiotic yogurt was associated with the abundance of SCFA-producing bacteria in the stool, such as Blautia, Roseburie, Bacteroides, Streptococcus, and Alloprevotella, as well as SCFA levels, including acetic acid, propionic acid, and butyric acid.

Probiotics have been found to reduce systolic blood pressure in rats by increasing TLR4 mRNA levels, increasing NADPH oxidase activity and endothelial nitric oxide synthase phosphorylation, and improving vascular pro-oxidative and pro-inflammatory states, changing the proportion of gut microbes (114). Kefir is a probiotic fermented dairy product derived from cereals (115). It has been found that kefir improves pathological changes in the small intestine, including restoration of Paneth cell mass and capsular myometrial thickness; normalization of circulating serum LPS levels, and reduction of TNF-α and IL-6 levels, thereby reducing the patient's blood pressure (116). In addition, Friques et al. (117) found that kefir also improved endothelial function in SHR, and the mechanism may be to restore ROS/NO imbalance and endothelial structure. Chronic use of probiotics increases intestinal integrity and reduces bacterial endotoxin entry into the circulation. Preventing intestinal dysbiosis in SHR patients prevented the development of endothelial dysfunction and hypertension (56). In addition, probiotics also suppress risk factors for hypertension by improving blood lipid levels, controlling body weight, and lowering body blood glucose levels (118–120). These studies suggest that probiotic therapy targeting gut microbiota has an important impact on the intervention of hypertension.

### 7.4 Antibiotic

The use of antibiotics is a recent development in the treatment of hypertension. It is important to consider individual differences during the treatment process, and not to overlook the adverse reactions and clinical effects of antibiotics. Sharma et al. (33) found that chemically modified tetracycline-3 (CMT-3) inhibition of the neuroinflammation of PVN can directly affect the gut microbiotaand its pathology to reduce hypertension. Galla's team treated young genetically hypertensive rats with amoxicillin to remodel the gut microbiota, particularly reducing succinate-producing microbiota, thereby lowering blood pressure, even after discontinuation of the drug (121). Doxycycline has been found to reduce the population of lactate-producing bacteria and plasma lactate levels, improve intestinal barrier integrity, inhibit endotoxemia, and reduce deoxycorticosterone acetate-induced hypertension in rats (122). Neomycin, minocycline, and vancomycin treated different types of hypertensive rats and it was found that all of the above antibiotics increased systolic blood pressure in Dahl salt-sensitive hypertensive rats, whereas minocycline and vancomycin lowered systolic blood pressure in SHR (123). Taken together, these studies strongly suggest the role of gut microbiota composition in hypertension, and an individualized approach to the use of antibiotics in hypertensive patients needs to be considered in the future.

However, some studies have shown that antibiotics can lead to reduced diversity of gut microbes and even adverse consequences of bacterial resistance (124, 125). The role of antibiotics in the treatment of hypertension remains controversial, and the selection of appropriate antibiotics requires further experimental validation.

### 7.5 Fecal microbiota transplantation

Replacement of the native microbiome of patients with microbiota-associated diseases with “healthy” microbial feces is called “fecal microbiota transplantation” (FMT) (126). By transplanting feces from hypertensive humans into germ-free mice, it was found that elevated blood pressure can be transferred through the microbiota (127). Adoptive transfer of fecal material from conventionally housed mice on a high-salt diet into germ-free mice makes them more susceptible to inflammation and hypertension (28). Kim et al. (128) transplanted fecal bacteria from healthy mice fed resveratrol into Ang II-induced hypertensive mice and found that systolic blood pressure (SBP) was decreased in hypertensive mice. However, the optimal FMT approach, including donor selection, screening, and preparation, has yet to be determined and therefore requires deeper exploration (37).

## 8 Conclusion and outlook

Increasing evidence suggests that the gut microbiota is associated with the development of hypertension and may be a novel target for hypertension treatment. The disruption of intestinal barrier function in hypertensive patients leads to bacterial translocation and endotoxin release into the blood, triggering a series of inflammatory and immune responses and aggravating hypertension, metabolites of gut microbiota can interact with hypertension, but the mechanism still needs further verification. Exercise, diet regulation, probiotic supplementation, antibacterial drug intervention, drug therapy, and fecal bacteria transplantation can effectively remodel the structure and richness of gut microbiota, increase the corresponding metabolites, and then relieve hypertension. However, the application of regulating the improvement of gut microbiota and its metabolites in the prevention, control, and treatment of hypertension is still in the animal experimental stage, and we need to continue to carry out more experiments to study its deeper mechanism and provide new ideas for the prevention and treatment of hypertension.

## References

[1] NCD Risk Factor Collaboration (NCD-RisC). Worldwide trends in hypertension prevalence and progress in treatment and control from 1990 to 2019: a pooled analysis of 1201 population-representative studies with 104 million participants. Lancet. (2021) 398(10304):957–80. 10.1016/S0140-6736(21)01330-1 34450083 PMC8446938

[2] Olsen MH Angell SY Asma S Boutouyrie P Burger D Chirinos JA A call to action and a lifecourse strategy to address the global burden of raised blood pressure on current and future generations: the lancet commission on hypertension. Lancet. (2016) 388(10060):2665–712. 10.1016/S0140-6736(16)31134-5 27671667

[3] Dai Y Shen Z Khachatryan LG Vadiyan DE Karampoor S Mirzaei R. Unraveling mechanistic insights into the role of microbiome in neurogenic hypertension: a comprehensive review. Pathol Res Pract. (2023) 249:154740. 10.1016/j.prp.2023.154740 37567034

[4] Luqman A Hassan A Ullah M Naseem S Ullah M Zhang L Role of the intestinal microbiome and its therapeutic intervention in cardiovascular disorder. Front Immunol. (2024) 15:1321395. 10.3389/fimmu.2024.1321395 38343539 PMC10853344

[5] Miao C Xu X Huang S Kong L He Z Wang Y The causality between gut microbiota and hypertension and hypertension-related complications: a bidirectional two-sample mendelian randomization analysis. Hellenic J Cardiol. (2024). 10.1016/j.hjc.2024.02.002 38336261

[6] Cox AJ West NP Cripps AW. Obesity, inflammation, and the gut microbiota. Lancet Diabetes Endocrinol. (2015) 3(3):207–15. 10.1016/S2213-8587(14)70134-2 25066177

[7] Grahnemo L Nethander M Coward E Gabrielsen ME Sree S Billod JM Cross-sectional associations between the gut microbe Ruminococcus gnavus and features of the metabolic syndrome. Lancet Diabetes Endocrinol. (2022) 10(7):481–3. 10.1016/S2213-8587(22)00113-9 35662399

[8] Kummen M Mayerhofer CCK Vestad B Broch K Awoyemi A. Gut microbiota signature in heart failure defined from profiling of 2 independent cohorts. J Am Coll Cardiol. (2018) 71(10):1184–6. 10.1016/j.jacc.2017.12.057 29519360

[9] Liu H Chen X Hu X Niu H Tian R Wang H Alterations in the gut microbiome and metabolism with coronary artery disease severity. Microbiome. (2019) 7(1):68. 10.1186/s40168-019-0683-9 31027508 PMC6486680

[10] Tang TWH Chen HC Chen CY Yen CYT Lin CJ Prajnamitra RP Loss of gut microbiota alters immune system composition and cripples postinfarction cardiac repair. Circulation. (2019) 139(5):647–59. 10.1161/CIRCULATIONAHA.118.035235 30586712

[11] Chen L Chen J Huang Y Wu Y Li J Ni W Changes of the gut microbiota composition and short chain fatty acid in patients with atrial fibrillation. PeerJ. (2023) 11:e16228. 10.7717/peerj.16228 38084144 PMC10710774

[12] Yan Q Gu Y Li X Yang W Jia L Chen C Alterations of the gut microbiome in hypertension. Front Cell Infect Microbiol. (2017) 7:381. 10.3389/fcimb.2017.00381 28884091 PMC5573791

[13] Sun S Lulla A Sioda M Winglee K Wu MC Jacobs DR Jr Gut microbiota composition and blood pressure. Hypertension. (2019) 73(5):998–1006. 10.1161/HYPERTENSIONAHA.118.12109 30905192 PMC6458072

[14] Yang T Santisteban MM Rodriguez V Li E Ahmari N Carvajal JM Gut dysbiosis is linked to hypertension. Hypertension. (2015) 65(6):1331–40. 10.1161/HYPERTENSIONAHA.115.05315 25870193 PMC4433416

[15] Virwani PD Qian G Hsu MSS Pijarnvanit T Cheung CN Chow YH Sex differences in association between gut microbiome and essential hypertension based on ambulatory blood pressure monitoring. Hypertension. (2023) 80(6):1331–42. 10.1161/HYPERTENSIONAHA.122.20752 37073724 PMC10191209

[16] Verhaar BJH Collard D Prodan A Levels JHM Zwinderman AH Bäckhed F Associations between gut microbiota, faecal short-chain fatty acids, and blood pressure across ethnic groups: the helius study. Eur Heart J. (2020) 41(44):4259–67. 10.1093/eurheartj/ehaa704 32869053 PMC7724641

[17] Guan XQ Wang CH Cheng P Fu LY Wu QJ Cheng G Effects of empagliflozin on gut microbiota in heart failure with a preserved ejection fraction: the design of a pragmatic randomized, open-label controlled trial (empagum). Drug Des Devel Ther. (2023) 17:1495–502. 10.2147/DDDT.S404479 37223722 PMC10202117

[18] Fukui H. Increased intestinal permeability and decreased barrier function: does it really influence the risk of inflammation? Inflamm Intest Dis. (2016) 1(3):135–45. 10.1159/000447252 29922669 PMC5988153

[19] Santisteban MM Qi Y Zubcevic J Kim S Yang T Shenoy V Hypertension-linked pathophysiological alterations in the gut. Circ Res. (2017) 120(2):312–23. 10.1161/CIRCRESAHA.116.309006 27799253 PMC5250568

[20] Adnan S Nelson JW Ajami NJ Venna VR Petrosino JF Bryan RM Jr Alterations in the gut microbiota can elicit hypertension in rats. Physiol Genomics. (2017) 49(2):96–104. 10.1152/physiolgenomics.00081.2016 28011881 PMC5336599

[21] Li L Zhong SJ Hu SY Cheng B Qiu H Hu ZX. Changes of gut microbiome composition and metabolites associated with hypertensive heart failure rats. BMC Microbiol. (2021) 21(1):141. 10.1186/s12866-021-02202-5 33952214 PMC8097775

[22] Jama HA Beale A Shihata WA Marques FZ. The effect of diet on hypertensive pathology: is there a link via gut microbiota-driven immunometabolism? Cardiovasc Res. (2019) 115(9):1435–47. 10.1093/cvr/cvz091 30951169

[23] Kim S Goel R Kumar A Qi Y Lobaton G Hosaka K Imbalance of gut microbiome and intestinal epithelial barrier dysfunction in patients with high blood pressure. Clin Sci (Lond). (2018) 132(6):701–18. 10.1042/CS20180087 29507058 PMC5955695

[24] Karbach SH Schonfelder T Brandao I Wilms E Hormann N Jackel S Gut microbiota promote angiotensin II-induced arterial hypertension and vascular dysfunction. J Am Heart Assoc. (2016) 5(9):e003698. 10.1161/JAHA.116.003698 27577581 PMC5079031

[25] Ayyaswamy S Shi H Zhang B Bryan RM Jr Durgan DJ. Obstructive sleep apnea-induced hypertension is associated with increased gut and neuroinflammation. J Am Heart Assoc. (2023) 12(11):e029218. 10.1161/JAHA.122.029218 37260032 PMC10381979

[26] Liu J Li T Wu H Shi H Bai J Zhao W Lactobacillus rhamnosus GG strain mitigated the development of obstructive sleep apnea-induced hypertension in a high salt diet via regulating tmao level and CD4(+) T cell induced-type I inflammation. Biomed Pharmacother. (2019) 112:108580. 10.1016/j.biopha.2019.01.041 30784906

[27] Wilck N Matus MG Kearney SM Olesen SW Forslund K Bartolomaeus H Salt-responsive gut commensal modulates T(H)17 axis and disease. Nature. (2017) 551(7682):585–9. 10.1038/nature24628 29143823 PMC6070150

[28] Ferguson JF Aden LA Barbaro NR Van Beusecum JP Xiao L Simmons AJ High dietary salt-induced dendritic cell activation underlies microbial dysbiosis-associated hypertension. JCI Insight. (2019) 5(13):e126241. 10.1172/jci.insight.126241 31162138 PMC6629246

[29] Richards EM Li J Stevens BR Pepine CJ Raizada MK. Gut microbiome and neuroinflammation in hypertension. Circ Res. (2022) 130(3):401–17. 10.1161/CIRCRESAHA.121.319816 35113664 PMC8852773

[30] Góralczyk-Bińkowska A Szmajda-Krygier D Kozłowska E. The microbiota-gut-brain axis in psychiatric disorders. Int J Mol Sci. (2022) 23(19):11245. 10.3390/ijms231911245 36232548 PMC9570195

[31] Erny D Hrabe de Angelis AL Jaitin D Wieghofer P Staszewski O David E Host microbiota constantly control maturation and function of microglia in the CNS. Nat Neurosci. (2015) 18(7):965–77. 10.1038/nn.4030 26030851 PMC5528863

[32] Zubcevic J Richards EM Yang T Kim S Sumners C Pepine CJ Impaired autonomic nervous system-microbiome circuit in hypertension. Circ Res. (2019) 125(1):104–16. 10.1161/CIRCRESAHA.119.313965 31219753 PMC6588177

[33] Sharma RK Yang T Oliveira AC Lobaton GO Aquino V Kim S Microglial cells impact gut microbiota and gut pathology in angiotensin II-induced hypertension. Circ Res. (2019) 124(5):727–36. 10.1161/CIRCRESAHA.118.313882 30612527 PMC6395495

[34] Santisteban MM Ahmari N Carvajal JM Zingler MB Qi Y Kim S Involvement of bone marrow cells and neuroinflammation in hypertension. Circ Res. (2015) 117(2):178–91. 10.1161/CIRCRESAHA.117.305853 25963715 PMC4490954

[35] Toral M Robles-Vera I de la Visitacion N Romero M Yang T Sanchez M Critical role of the interaction gut microbiota—sympathetic nervous system in the regulation of blood pressure. Front Physiol. (2019) 10:231. 10.3389/fphys.2019.00231 30930793 PMC6423906

[36] Robles-Vera I de la Visitacion N Toral M Sanchez M Gomez-Guzman M Jimenez R Mycophenolate mediated remodeling of gut microbiota and improvement of gut-brain axis in spontaneously hypertensive rats. Biomed Pharmacother. (2021) 135:111189. 10.1016/j.biopha.2020.111189 33388596

[37] Verhaar BJH Prodan A Nieuwdorp M Muller M. Gut microbiota in hypertension and atherosclerosis: a review. Nutrients. (2020) 12(10):2982. 10.3390/nu12102982 33003455 PMC7601560

[38] Chen M Miao G Zhang Y Umans JG Lee ET Howard BV Longitudinal lipidomic profile of hypertension in American Indians: findings from the strong heart family study. Hypertension. (2023) 80(8):1771–83. 10.1161/HYPERTENSIONAHA.123.21144 37334699 PMC10526703

[39] Kulkarni H Mamtani M Blangero J Curran JE. Lipidomics in the study of hypertension in metabolic syndrome. Curr Hypertens Rep. (2017) 19(1):7. 10.1007/s11906-017-0705-6 28168678

[40] Wang T Liu L Deng J Jiang Y Yan X Liu W. Analysis of the mechanism of action of quercetin in the treatment of hyperlipidemia based on metabolomics and intestinal flora. Food Funct. (2023) 14(4):2112–27. 10.1039/D2FO03509J 36740912

[41] Ding H Zhang H Lu Y Jiang X Liu Q Hu Y Effects of the polypeptide from peanut meal mixed fermentation on lipid metabolism and intestinal flora of hyperlipidemic mice. J Sci Food Agric. (2023) 103(9):4351–9. 10.1002/jsfa.12500 36782346

[42] Wang Z Yao W Sun Y Han Y Chen X Gong P Eucommia bark/leaf extract improves lipid metabolism disorders by affecting intestinal microbiota and microbiome-host interaction in HFD mice. J Agric Food Chem. (2023) 71(7):3297–314. 10.1021/acs.jafc.2c07239 36753681

[43] Aktaa S Gale CP Brida M Giannakoulas G Kovacs G Adir Y European society of cardiology quality indicators for the care and outcomes of adults with pulmonary arterial hypertension. Developed in collaboration with the heart failure association of the European Society of Cardiology. Eur J Heart Fail. (2023) 25(4):469–77. 10.1002/ejhf.2830 36924171

[44] Louca P Menni C PadmanabhaN S. Genomic determinants of hypertension with a focus on metabolomics and the gut microbiome. Am J Hypertens. (2020) 33(6):473–81. 10.1093/ajh/hpaa022 32060494 PMC7241940

[45] O'Donnell JA Zheng T Meric G Marques FZ. The gut microbiome and hypertension. Nat Rev Nephrol. (2023) 19(3):153–67. 10.1038/s41581-022-00654-0 36631562

[46] Kim M Huda MN Bennett BJ. Sequence meets function-microbiota and cardiovascular disease. Cardiovasc Res. (2022) 118(2):399–412. 10.1093/cvr/cvab030 33537709 PMC8803075

[47] Parada Venegas D De la Fuente MK Landskron G González MJ Quera R Dijkstra G Short chain fatty acids (scfas)-mediated gut epithelial and immune regulation and its relevance for inflammatory bowel diseases. Front Immunol. (2019) 10:277. 10.3389/fimmu.2019.00277 30915065 PMC6421268

[48] Xu J Moore BN Pluznick JL. Short-chain fatty acid receptors and blood pressure regulation: council on hypertension mid-career award for research excellence 2021. Hypertension. (2022) 79(10):2127–37. 10.1161/HYPERTENSIONAHA.122.18558 35912645 PMC9458621

[49] Calderon-Perez L Gosalbes MJ Yuste S Valls RM Pedret A Llaurado E Gut metagenomic and short chain fatty acids signature in hypertension: a cross-sectional study. Sci Rep. (2020) 10(1):6436. 10.1038/s41598-020-63475-w 32296109 PMC7160119

[50] Huart J Cirillo A Taminiau B Descy J Saint-Remy A Daube G Human stool metabolome differs upon 24 h blood pressure levels and blood pressure dipping status: a prospective longitudinal study. Metabolites. (2021) 11(5):282. 10.3390/metabo11050282 33946722 PMC8146767

[51] de la Cuesta-Zuluaga J Mueller NT Álvarez-Quintero R Velásquez-Mejía EP Sierra JA Corrales-Agudelo V Higher fecal short-chain fatty acid levels are associated with gut microbiome dysbiosis, obesity, hypertension and cardiometabolic disease risk factors. Nutrients. (2018) 11(1):51. 10.3390/nu11010051 30591685 PMC6356834

[52] Pluznick JL. Microbial short-chain fatty acids and blood pressure regulation. Curr Hypertens Rep. (2017) 19(4):25. 10.1007/s11906-017-0722-5 28315048 PMC5584783

[53] Nogal A Valdes AM Menni C. The role of short-chain fatty acids in the interplay between gut microbiota and diet in cardio-metabolic health. Gut Microbes. (2021) 13(1):1–24. 10.1080/19490976.2021.1897212 33764858 PMC8007165

[54] Kaye DM Shihata WA Jama HA Tsyganov K Ziemann M Kiriazis H Deficiency of prebiotic fiber and insufficient signaling through gut metabolite-sensing receptors leads to cardiovascular disease. Circulation. (2020) 141(17):1393–403. 10.1161/CIRCULATIONAHA.119.043081 32093510

[55] Pluznick J. A novel scfa receptor, the microbiota, and blood pressure regulation. Gut Microbes. (2014) 5(2):202–7. 10.4161/gmic.27492 24429443 PMC4063845

[56] Robles-Vera I Toral M de la Visitacion N Sanchez M Gomez-Guzman M Romero M Probiotics prevent dysbiosis and the rise in blood pressure in genetic hypertension: role of short-chain fatty acids. Mol Nutr Food Res. (2020) 64(6):e1900616. 10.1002/mnfr.201900616 31953983

[57] Moleón J González-Correa C Miñano S Robles-Vera I de la Visitación N Barranco AM Protective effect of microbiota-derived short chain fatty acids on vascular dysfunction in mice with systemic lupus erythematosus induced by toll like receptor 7 activation. Pharmacol Res. (2023) 198:106997. 10.1016/j.phrs.2023.106997 37972724

[58] Bartolomaeus H Balogh A Yakoub M Homann S Markó L Höges S Short-chain fatty acid propionate protects from hypertensive cardiovascular damage. Circulation. (2019) 139(11):1407–21. 10.1161/CIRCULATIONAHA.118.036652 30586752 PMC6416008

[59] Jin J Gao L Zou X Zhang Y Zheng Z Zhang X Gut dysbiosis promotes preeclampsia by regulating macrophages and trophoblasts. Circ Res. (2022) 131(6):492–506. 10.1161/CIRCRESAHA.122.320771 35950704

[60] Wang L Zhu Q Lu A Liu X Zhang L Xu C Sodium butyrate suppresses angiotensin II-induced hypertension by inhibition of renal (pro)renin receptor and intrarenal renin-angiotensin system. J Hypertens. (2017) 35(9):1899–908. 10.1097/HJH.0000000000001378 28509726 PMC11157961

[61] Tang WHW Li DY Hazen SL. Dietary metabolism, the gut microbiome, and eart failure. Nat Rev Cardiol. (2018) 16(3):137–54. 10.1038/s41569-018-0108-7 PMC6377322 30410105

[62] Constantino-Jonapa LA Espinoza-Palacios Y Escalona-Montaño AR Hernández-Ruiz P Amezcua-Guerra LM Amedei A Contribution of trimethylamine N-oxide (tmao) to chronic inflammatory and degenerative diseases. Biomedicines. (2023) 11(2):431. 10.3390/biomedicines11020431 36830968 PMC9952918

[63] Yu ZL Zhang LY Jiang XM Xue CH Chi N Zhang TT Effects of dietary choline, betaine, and L-carnitine on the generation of trimethylamine-N-oxide in healthy mice. J Food Sci. (2020) 85(7):2207–15. 10.1111/1750-3841.15186 32572979

[64] Witkowski M Weeks TL Hazen SL. Gut microbiota and cardiovascular disease. Circ Res. (2020) 127(4):553–70. 10.1161/CIRCRESAHA.120.316242 32762536 PMC7416843

[65] Jiang S Shui Y Cui Y Tang C Wang X Qiu X Gut microbiota dependent trimethylamine N-oxide aggravates angiotensin II-induced hypertension. Redox Biol. (2021) 46:102115. 10.1016/j.redox.2021.102115 34474396 PMC8408632

[66] Ufnal M Jazwiec R Dadlez M Drapala A Sikora M Skrzypecki J. Trimethylamine-N-oxide: a carnitine-derived metabolite that prolongs the hypertensive effect of angiotensin II in rats. Can J Cardiol. (2014) 30(12):1700–5. 10.1016/j.cjca.2014.09.010 25475471

[67] Liu M Han Q Yang J. Trimethylamine-N-oxide (tmao) increased aquaporin-2 expression in spontaneously hypertensive rats. Clin Exp Hypertens. (2019) 41(4):312–22. 10.1080/10641963.2018.1481420 29985655

[68] Zhen J Zhou Z He M Han HX Lv EH Wen PB The gut microbial metabolite trimethylamine N-oxide and cardiovascular diseases. Front Endocrinol (Lausanne). (2023) 14:1085041. 10.3389/fendo.2023.1085041 36824355 PMC9941174

[69] Li D Lu Y Yuan S Cai X He Y Chen J Gut microbiota-derived metabolite trimethylamine-N-oxide and multiple health outcomes: an umbrella review and updated meta-analysis. Am J Clin Nutr. (2022) 116(1):230–43. 10.1093/ajcn/nqac074 35348578 PMC9257469

[70] Han JM Guo L Chen XH Xie Q Song XY Ma YL. Relationship between trimethylamine N-oxide and the risk of hypertension in patients with cardiovascular disease: a meta-analysis and dose-response relationship analysis. Medicine (Baltimore). (2024) 103(1):e36784. 10.1097/MD.0000000000036784 38181288 PMC10766215

[71] Ge X Zheng L Zhuang R Yu P Xu Z Liu G The gut microbial metabolite trimethylamine N-oxide and hypertension risk: a systematic review and dose-response meta-analysis. Adv Nutr. (2020) 11(1):66–76. 10.1093/advances/nmz064 31269204 PMC7442397

[72] Nie J Xie L Zhao BX Li Y Qiu B Zhu F Serum trimethylamine N-oxide concentration is positively associated with first stroke in hypertensive patients. Stroke. (2018) 49(9):2021–8. 10.1161/STROKEAHA.118.021997 30354996

[73] Huc T Drapala A Gawrys M Konop M Bielinska K Zaorska E Chronic, low-dose tmao treatment reduces diastolic dysfunction and heart fibrosis in hypertensive rats. Am J Physiol Heart Circ Physiol. (2018) 315(6):H1805–20. 10.1152/ajpheart.00536.2018 30265149

[74] Rathinam VAK Zhao Y Shao F. Innate immunity to intracellular LPS. Nat Immunol. (2019) 20(5):527–33. 10.1038/s41590-019-0368-3 30962589 PMC7668400

[75] Wang Z Zhao Y. Gut microbiota derived metabolites in cardiovascular health and disease. Protein Cell. (2018) 9(5):416–31. 10.1007/s13238-018-0549-0 29725935 PMC5960473

[76] Zhan L Zheng J Meng J Fu D Pang L Ji C. Toll-like receptor 4 deficiency alleviates lipopolysaccharide-induced intestinal barrier dysfunction. Biomed Pharmacother. (2022) 155:113778. 10.1016/j.biopha.2022.113778 36271559

[77] Grylls A Seidler K Neil J. Link between microbiota and hypertension: focus on LPS/TLR4 pathway in endothelial dysfunction and vascular inflammation, and therapeutic implication of probiotics. Biomed Pharmacother. (2021) 137:111334. 10.1016/j.biopha.2021.111334 33556874

[78] Masson GS Nair AR Dange RB Silva-Soares PP Michelini LC Francis J. Toll-like receptor 4 promotes autonomic dysfunction, inflammation and microglia activation in the hypothalamic paraventricular nucleus: role of endoplasmic reticulum stress. PLoS One. (2015) 10(3):e0122850. 10.1371/journal.pone.0122850 25811788 PMC4374971

[79] Dange RB Agarwal D Teruyama R Francis J. Toll-like receptor 4 inhibition within the paraventricular nucleus attenuates blood pressure and inflammatory response in a genetic model of hypertension. J Neuroinflammation. (2015) 12:31. 10.1186/s12974-015-0242-7 25879545 PMC4337244

[80] Wang J Gu X Yang J Wei Y Zhao Y. Gut microbiota dysbiosis and increased plasma LPS and tmao levels in patients with preeclampsia. Front Cell Infect Microbiol. (2019) 9:409. 10.3389/fcimb.2019.00409 31850241 PMC6901393

[81] Poll BG Cheema MU Pluznick JL. Gut microbial metabolites and blood pressure regulation: focus on scfas and tmao. Physiology (Bethesda). (2020) 35(4):275–84. 10.1152/physiol.00004.2020 32490748 PMC7474256

[82] Tomasova L Dobrowolski L Jurkowska H Wróbel M Huc T Ondrias K Intracolonic hydrogen sulfide lowers blood pressure in rats. Nitric Oxide. (2016) 60:50–8. 10.1016/j.niox.2016.09.007 27667183

[83] Weber GJ Pushpakumar S Tyagi SC Sen U. Homocysteine and hydrogen sulfide in epigenetic, metabolic and microbiota related renovascular hypertension. Pharmacol Res. (2016) 113(Pt A):300–12. 10.1016/j.phrs.2016.09.002 27602985 PMC5107119

[84] Donertas Ayaz B Zubcevic J. Gut microbiota and neuroinflammation in pathogenesis of hypertension: a potential role for hydrogen sulfide. Pharmacol Res. (2020) 153:104677. 10.1016/j.phrs.2020.104677 32023431 PMC7056572

[85] Tomasova L Konopelski P Ufnal M. Gut bacteria and hydrogen sulfide: the new old players in circulatory system homeostasis. Molecules. (2016) 21(11):1558. 10.3390/molecules21111558 27869680 PMC6273628

[86] Al-Magableh MR Kemp-Harper BK Hart JL. Hydrogen sulfide treatment reduces blood pressure and oxidative stress in angiotensin II-induced hypertensive mice. Hypertens Res. (2015) 38(1):13–20. 10.1038/hr.2014.125 25099489

[87] Xiao L Dong JH Teng X Jin S Xue HM Liu SY Hydrogen sulfide improves endothelial dysfunction in hypertension by activating peroxisome proliferator-activated receptor delta/endothelial nitric oxide synthase signaling. J Hypertens. (2018) 36(3):651–65. 10.1097/HJH.0000000000001605 29084084

[88] Marques FZ Nelson E Chu PY Horlock D Fiedler A Ziemann M High-fiber diet and acetate supplementation change the gut microbiota and prevent the development of hypertension and heart failure in hypertensive mice. Circulation. (2017) 135(10):964–77. 10.1161/CIRCULATIONAHA.116.024545 27927713

[89] Estruch R Ros E Salas-Salvado J Covas MI Corella D Aros F Primary prevention of cardiovascular disease with a Mediterranean diet supplemented with extra-virgin olive oil or nuts. N Engl J Med. (2018) 378(25):e34. 10.1056/NEJMoa1800389 29897866

[90] Merra G Noce A Marrone G Cintoni M Tarsitano MG Capacci A Influence of Mediterranean diet on human gut microbiota. Nutrients. (2020) 13(1):7. 10.3390/nu13010007 33375042 PMC7822000

[91] Choo JM Murphy KJ Wade AT Wang Y Bracci EL Davis CR Interactions between Mediterranean diet supplemented with dairy foods and the gut microbiota influence cardiovascular health in an Australian population. Nutrients. (2023) 15(16):3645. 10.3390/nu15163645 37630835 PMC10459086

[92] Li Y Salih Ibrahim RM Chi HL Xiao T Xia WJ Li HB Altered gut microbiota is involved in the anti-hypertensive effects of vitamin C in spontaneously hypertensive rat. Mol Nutr Food Res. (2021) 65(7):e2000885. 10.1002/mnfr.202000885 33547879

[93] Calderón-Pérez L Llauradó E Companys J Pla-Pagà L Pedret A Rubió L Interplay between dietary phenolic compound intake and the human gut microbiome in hypertension: a cross-sectional study. Food Chem. (2021) 344:128567. 10.1016/j.foodchem.2020.128567 33203597

[94] Shi H Zhang B Abo-Hamzy T Nelson JW Ambati CSR Petrosino JF Restructuring the gut microbiota by intermittent fasting lowers blood pressure. Circ Res. (2021) 128(9):1240–54. 10.1161/CIRCRESAHA.120.318155 33596669 PMC8085162

[95] Xia WJ Xu ML Yu XJ Du MM Li XH Yang T Antihypertensive effects of exercise involve reshaping of gut microbiota and improvement of gut-brain axis in spontaneously hypertensive rat. Gut Microbes. (2021) 13(1):1–24. 10.1080/19490976.2020.1854642 PMC7781639 33382364

[96] Wu S Zheng C Liu N Deng T Wang J Qi L Liuzijue training improves hypertension and modulates gut microbiota profile. Front Cardiovasc Med. (2023) 10:1075084. 10.3389/fcvm.2023.1075084 36760555 PMC9905721

[97] Barton W Penney NC Cronin O Garcia-Perez I Molloy MG Holmes E The microbiome of professional athletes differs from that of more sedentary subjects in composition and particularly at the functional metabolic level. Gut. (2018) 67(4):625–33. 10.1136/gutjnl-2016-313627 28360096

[98] Yang T Aquino V Lobaton GO Li H Colon-Perez L Goel R Sustained captopril-induced reduction in blood pressure is associated with alterations in gut-brain axis in the spontaneously hypertensive rat. J Am Heart Assoc. (2019) 8(4):e010721. 10.1161/JAHA.118.010721 30755073 PMC6405665

[99] Wu D Tang X Ding L Cui J Wang P Du X Candesartan attenuates hypertension-associated pathophysiological alterations in the gut. Biomed Pharmacother. (2019) 116:109040. 10.1016/j.biopha.2019.109040 31170664

[100] Xiong Y He Y Chen Z Wu T Xiong Y Peng Y Lactobacillus induced by irbesartan on spontaneously hypertensive rat contribute to its antihypertensive effect. J Hypertens. (2024) 42(3):460–70. 10.1097/HJH.0000000000003613 38009301

[101] Robles-Vera I Toral M de la Visitación N Sánchez M Gómez-Guzmán M Muñoz R Changes to the gut microbiota induced by losartan contributes to its antihypertensive effects. Br J Pharmacol. (2020) 177(9):2006–23. 10.1111/bph.14965 31883108 PMC7161554

[102] Dong S Liu Q Zhou X Zhao Y Yang K Li L Effects of losartan, atorvastatin, and aspirin on blood pressure and gut microbiota in spontaneously hypertensive rats. Molecules. (2023) 28(2):612. 10.3390/molecules28020612 36677668 PMC9860566

[103] González-Correa C Moleón J Miñano S Robles-Vera I Toral M Martín-Morales N Mineralocorticoid receptor blockade improved gut microbiota dysbiosis by reducing gut sympathetic tone in spontaneously hypertensive rats. Biomed Pharmacother. (2023) 158:114149. 10.1016/j.biopha.2022.114149 36566524

[104] Wu D Ding L Tang X Wang W Chen Y Zhang T. Baicalin protects against hypertension-associated intestinal barrier impairment in part through enhanced microbial production of short-chain fatty acids. Front Pharmacol. (2019) 10:1271. 10.3389/fphar.2019.01271 31719823 PMC6826474

[105] Wang Z Wu F Zhou Q Qiu Y Zhang J Tu Q Berberine improves vascular dysfunction by inhibiting trimethylamine-N-oxide via regulating the gut Microbiota in angiotensin II-induced hypertensive mice. Front Microbiol. (2022) 13:814855. 10.3389/fmicb.2022.814855 35350612 PMC8957906

[106] Wu J Nakashima S Nakamura S Matsuda H. Effects of sanoshashinto on left ventricular hypertrophy and gut microbiota in spontaneously hypertensive rats. J Nat Med. (2020) 74(2):482–6. 10.1007/s11418-020-01387-9 31956959

[107] Li H-B Xu M-L Du M-M Yu X-J Bai J Xia W-J Curcumin ameliorates hypertension via gut-brain communication in spontaneously hypertensive rat. Toxicol Appl Pharmacol. (2021) 429:115701. 10.1016/j.taap.2021.115701 34453990

[108] Su J Wang Y Yan M He Z Zhou Y Xu J The beneficial effects of Polygonatum sibiricum red. Superfine powder on metabolic hypertensive rats via gut-derived LPS/TLR4 pathway inhibition. Phytomedicine. (2022) 106:154404. 10.1016/j.phymed.2022.154404 36075182

[109] Yang Z Lin S Liu Y Song Z Ge Z Fan Y Targeting intestinal microecology: potential intervention strategies of traditional Chinese medicine for managing hypertension. Front Pharmacol. (2023) 14:1171119. 10.3389/fphar.2023.1171119 37324472 PMC10264781

[110] Qi D Nie XL Zhang JJ. The effect of probiotics supplementation on blood pressure: a systemic review and meta-analysis. Lipids Health Dis. (2020) 19(1):79. 10.1186/s12944-020-01259-x 32334580 PMC7183137

[111] Khalesi S Sun J Buys N Jayasinghe R. Effect of probiotics on blood pressure: a systematic review and meta-analysis of randomized, controlled trials. Hypertension. (2014) 64(4):897–903. 10.1161/HYPERTENSIONAHA.114.03469 25047574

[112] Wang XL Chen WJ Jin R Xu X Wei J Huang H Engineered probiotics Clostridium butyricum-pMTL007-GLP-1 improves blood pressure via producing GLP-1 and modulating gut microbiota in spontaneous hypertension rat models. Microb Biotechnol. (2023) 16(4):799–812. 10.1111/1751-7915.14196 36528874 PMC10034621

[113] Kong CY Li ZM Mao YQ Chen HL Hu W Han B Probiotic yogurt blunts the increase of blood pressure in spontaneously hypertensive rats via remodeling of the gut microbiota. Food Funct. (2021) 12(20):9773–83. 10.1039/D1FO01836A 34494630

[114] Gomez-Guzman M Toral M Romero M Jimenez R Galindo P Sanchez M Antihypertensive effects of probiotics Lactobacillus strains in spontaneously hypertensive rats. Mol Nutr Food Res. (2015) 59(11):2326–36. 10.1002/mnfr.201500290 26255877

[115] Silva-Cutini MA Almeida SA Nascimento AM Abreu GR Bissoli NS Lenz D Long-term treatment with kefir probiotics ameliorates cardiac function in spontaneously hypertensive rats. J Nutr Biochem. (2019) 66:79–85. 10.1016/j.jnutbio.2019.01.006 30776608

[116] de Almeida Silva M Mowry FE Peaden SC Andrade TU Biancardi VC. Kefir ameliorates hypertension via gut–brain mechanisms in spontaneously hypertensive rats. J Nutr Biochem. (2020) 77:108318. 10.1016/j.jnutbio.2019.108318 31923755

[117] Friques AG Arpini CM Kalil IC Gava AL Leal MA Porto ML Chronic administration of the probiotic kefir improves the endothelial function in spontaneously hypertensive rats. J Transl Med. (2015) 13:390. 10.1186/s12967-015-0759-7 26715471 PMC4696190

[118] Brusaferro A Cozzali R Orabona C Biscarini A Farinelli E Cavalli E Is it time to use probiotics to prevent or treat obesity? Nutrients. (2018) 10(11):1613. 10.3390/nu10111613 30388851 PMC6266556

[119] Yuan L Li Y Chen M Xue L Wang J Ding Y Effects of probiotics on hypertension. Appl Microbiol Biotechnol. (2023) 107(4):1107–17. 10.1007/s00253-023-12369-8 36646911

[120] Mu J Guo X Zhou Y Cao G. The effects of probiotics/synbiotics on glucose and lipid metabolism in women with gestational diabetes Mellitus: a meta-analysis of randomized controlled trials. Nutrients. (2023) 15(6):1375. 10.3390/nu15061375 36986107 PMC10056932

[121] Galla S Chakraborty S Cheng X Yeo JY Mell B Chiu N Exposure to amoxicillin in early life is associated with changes in gut microbiota and reduction in blood pressure: findings from a study on rat dams and offspring. J Am Heart Assoc. (2020) 9(2):e014373. 10.1161/JAHA.119.014373 31928175 PMC7033837

[122] Robles-Vera I de la Visitación N Toral M Sánchez M Romero M Gómez-Guzmán M Changes in gut microbiota induced by doxycycline influence in vascular function and development of hypertension in doca-salt rats. Nutrients. (2021) 13(9):2971. 10.3390/nu13092971 34578849 PMC8464928

[123] Galla S Chakraborty S Cheng X Yeo J Mell B Zhang H Disparate effects of antibiotics on hypertension. Physiol Genomics. (2018) 50(10):837–45. 10.1152/physiolgenomics.00073.2018 30095376 PMC6230872

[124] Santacroce L Di Domenico M Montagnani M Jirillo E. Antibiotic resistance and microbiota response. Curr Pharm Des. (2023) 29(5):356–64. 10.2174/1381612829666221219093450 36537602

[125] Dahiya D Nigam PS. Antibiotic-therapy-induced gut dysbiosis affecting gut microbiota-brain axis and cognition: restoration by intake of probiotics and synbiotics. Int J Mol Sci. (2023) 24(4):3074. 10.3390/ijms24043074 36834485 PMC9959899

[126] Sanchez-Rodriguez E Egea-Zorrilla A Plaza-Díaz J Aragón-Vela J Muñoz-Quezada S Tercedor-Sánchez L The gut microbiota and its implication in the development of atherosclerosis and related cardiovascular diseases. Nutrients. (2020) 12(3):605. 10.3390/nu12030605 32110880 PMC7146472

[127] Li J Zhao F Wang Y Chen J Tao J Tian G Gut microbiota dysbiosis contributes to the development of hypertension. Microbiome. (2017) 5(1):14. 10.1186/s40168-016-0222-x 28143587 PMC5286796

[128] Kim TT Parajuli N Sung MM Bairwa SC Levasseur J Soltys CM Fecal transplant from resveratrol-fed donors improves glycaemia and cardiovascular features of the metabolic syndrome in mice. Am J Physiol Endocrinol Metab. (2018) 315(4):E511–9. 10.1152/ajpendo.00471.2017 29870676

